# A Presentation of Neisseria meningitidis in a Patient Taking Adalimumab as Immunosuppressive Therapy for Hidradenitis Suppurativa

**DOI:** 10.7759/cureus.68628

**Published:** 2024-09-04

**Authors:** Jacob W Bolling, Aaron D Holley, Cleon Rogers

**Affiliations:** 1 Medicine, Christ Health Center, Edward Via College of Osteopathic Medicine, Birmingham, USA; 2 Internal Medicine, Christ Health Center, Birmingham, USA

**Keywords:** tnf-alpha inhibitors, humira, immunosuppressive therapy, adalimumab, atypical presentation, neisseria meningitidis

## Abstract

*Neisseria meningitidis* is common within the human population. Most patients with *N. meningitidis* colonization are asymptomatic, but invasive disease can result in meningitis, fulminant septicemia, and disseminated intravascular coagulation. This case report describes a patient who presented with symptoms of sepsis and was later diagnosed with *N. meningitidis*. The cause of her infection was believed to be immunosuppression from adalimumab, which she was taking for systemic hidradenitis suppurativa.

## Introduction

*Neisseria meningititidis* is an encapsulated, aerobic, gram-negative diplococcus and is the world’s leading cause of meningitis [1]. It is carried within roughly 10% to 35% of the human population, with the rate increasing to nearly 100% in people who live in close contact with each other, such as college students and military recruits [2]. Carriage can last from days to months [1]. Once acquired, a patient could either be asymptomatic, have only pharyngitis, or have an invasive disease [1]. Globally, there are an estimated 1.2 million cases of *N. meningitidis* each year, with an estimated 135,000 deaths [1]. Fulminant septicemia carries a 55% mortality rate [2].

This bacterium has multiple ways to evade the immune system. This includes antigenic variation, allelic exchange of gene fragments, intragenomic recombination, and varied levels of gene expressivity [2]. It enters the vasculature by either transcellular or paracellular routes [3]. In the transcellular route, pili make the initial contact with epithelial cells. From there, Opa proteins on bacterial pili bind to carcinoembryonic antigen-related cell adhesion molecules (CEACAMs) and heparan sulfate proteoglycans (HSPGs) on the host cell membrane. This results in a cascade of events leading to *N. meningitidis* internalizing within the host epithelial cell [2]. Bacterial cells traverse to the basolateral surface and bind to unknown receptors to facilitate transportation into the host vasculature system [2]. In the vasculature, they can resist blood flow velocities. This allows them to remain in place to form colonies [3]. Once at the blood-brain barrier, it is not known whether they cross over via a damaged blood-brain barrier through fenestrations or by transcytosis through an intact barrier [2]. Once in the central nervous system, bacteria replicate exponentially due to a lack of complement and immunoglobulins [3].

The innate immune system is the body's first line of defense against *N. meningitidis*. However, *N. meningitidis *escapes polymorphonuclear neutrophils of the innate immune system by uptaking L-glutamate and converting it to glutathione, thus helping to prevent an oxidative burst [3]. Once in the bloodstream, lipopolysaccharides in the gram-negative cell wall induce an inflammatory response through their interactions with toll-like receptor 4 (TLR4) [3]. Inflammatory damage causes a loss of capillary integrity. This results in increased permeability, hemorrhage, thrombi formation, and the eventual development of purpura fulminans [3].

Risk factors associated with acquiring *N. meningitidis *include being a child younger than one, an individual between 16 and 23, or an adult older than 65; having a complement component deficiency, functional and anatomic asplenia, or HIV; and being on immunosuppressive medications, especially eculizumab and ravulizumab [4]. *Neisseria meningititidis* is spread through respiratory and throat secretions [4]. It is most likely to be spread to those who live together or share close contact [4]. Typical signs and symptoms include fever, stiff neck, headache, confusion, light sensitivity, and nausea and vomiting [4].

Once a diagnosis of *N. meningitidis* is suspected, broad-spectrum antibiotics should be started immediately. For children, cefotaxime should be started with weight-based dosing [5]. In adults, either ceftriaxone or cefotaxime should be started [5]. In the event of shock, supportive measures with IV crystalloid, vasopressors, steroids, insulin drip, mechanical ventilation, dialysis, venous thromboembolism prophylaxis, and stress ulcer prophylaxis should be initiated based on patient need [5]. An *N. meningitidis* infection prevention with a conjugated meningococcal vaccine is recommended for those aged between 11 and 18, military recruits, and college students [5]. Vaccination is also recommended for those with anatomic or functional asplenia, complement component deficiencies, use of complement inhibitors, occupational exposure, and those associated with an outbreak [5]. For those exposed, antibiotic prophylaxis with either rifampin, ciprofloxacin, or ceftriaxone could be initiated within 24 hours of exposure [5].

## Case presentation

A 42-year-old female with a past medical history of embolic stroke with left-sided deficits, pulmonary embolism, type 2 diabetes, hidradenitis suppurativa, and pustular psoriasis presented to the emergency department by ambulance for a decrease in oral intake and worsening mental status. Medications included amitriptyline, atorvastatin, baclofen, apixiban, fluoxetine, adalimumab, and metformin.

**Figure 1:**
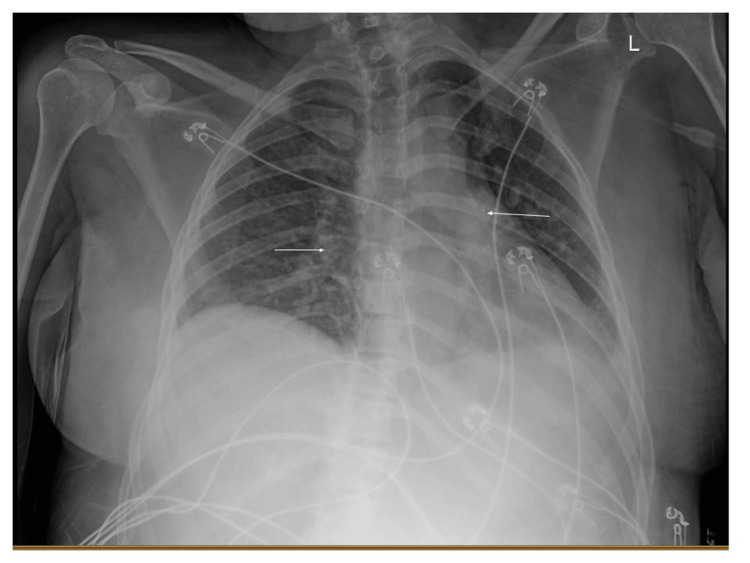
Initial chest X-ray The arrows point to bilateral patchy infiltrates.

A physical exam in the emergency department showed an ill-appearing female in mild distress. She was only mildly responsive to noxious stimuli. She had no nuchal rigidity and had a negative Kernig and Brudzinski sign. The remaining physical exam was unremarkable. Vitals showed a temperature of 38.7°C, a heart rate of 117 bpm, a respiratory rate of 22 br/min, and a blood pressure of 76/50. The initial chest X-ray showed bilateral patchy infiltrates (Figure 1). Initial labs are shown in Tables 1-4. Due to concern for septic shock, blood cultures were drawn, two liters of IV sodium chloride 0.9% were given, and vancomycin and piperacillin/tazobactam were started. The patient was then admitted to the ICU for further management.

**Table 1:** Initial venous blood gas values

Labs	Patient lab values	Normal reference lab values
Venous pH	7.454	7.31-7.41
Venous pC02	27.3 mmHg	41-51 mmHg
Venous pO2	104.5 mmHg	35-40 mmHg
Venous HCO3	18.7 mEq/l	24-28 mEq/l

**Table 2:** Initial complete blood count

Labs	Patient lab values	Normal reference lab values
White blood cells	5,500 cells/mcl	4,500-10,000 cells/mcl
Hemoglobin	10.5 g/dl	12.1-15.1 g/dl
Hematocrit	31.9%	36.1-44.3%
Platelets	177 K/mcl	150-400 K/mcl
Neutrophils	92.7%	50-70%
Lymphocytes	4.3%	18-42%
Monocytes	1.4%	3.5-9.0%
Eosinophils	0.5%	1-3%
Basophils	1.1%	0-2%

**Table 3:** Initial coagulation studies

Labs	Patient lab values	Normal reference lab values
Prothrombin time	19.1 seconds	10-13 seconds
International normalized ratio	1.70	0.8-1.2
Partial thromboplastin time	36.9 seconds	25-36 seconds

**Table 4:** Initial comprehensive metabolic panel

Labs	Patient lab values	Normal reference lab values
Sodium	135 mEq/l	135-145 mEq/l
Potassium	2.6 mEq/l	3.5-5 mEq/l
Chloride	104 mEq/l	95-105 mEq/l
CO2	16 mEq/l	23-29 mEq/l
Blood urea nitrogen	18 mg/dl	5-20 mg/dl
Glucose	176 mg/dl	70-100 mg/dl
Creatinine	.97 mg/dl	0.6-1.2 mg/dl
Alanine transaminase	13 U/l	7-60 U/l
Aspartate aminotransferase	16 U/l	9-40 U/l

Shortly after initial resuscitation, a physical exam showed the patient to be fully alert and oriented. She remained tachycardic and tachypnic, but she was now afebrile and normotensive on IV vasopressors. A CT scan of the head and brain was significant for findings consistent with chronic ischemia but showed no additional acute intracranial abnormalities. A CT scan of the abdomen and pelvis showed urinary bladder wall thickening, potential hepatic steatosis, cholelithiasis, and a moderate fecal burden. Subsequent labs showed an elevated WBC and an elevation of liver enzymes (Table 5). Preliminary blood cultures showed gram-positive cocci. Non-culture polymerase chain reaction (PCR) microbiology results were positive for *N. meningitidis*. Piperacillin/tazobactam was stopped at this time.

**Table 5:** Significant labs from day one of hospital admission

Labs	Patient lab values	Normal reference lab values
White blood cell	15.2 cells/mcl	4,500-10,000 cells/mcl
Aspartate transferase	640 U/l	9-40 U/l
Alanine transaminase	660 U/l	7-60 U/l

Vancomycin was continued, and high-dose ceftriaxone was initiated. The Department of Infectious Diseases was consulted to assist with further management. No lumbar puncture was performed as the patient’s platelets dropped from 177 K/mcL to 62 K/mcL. It was determined that a lumbar puncture was a high-risk procedure and would not change management.

On day two of hospital admission, the patient remained afebrile. Her tachycardia and tachypnea also resolved at this time. Blood pressure remained stable, and pressors were weaned off. Repeat labs showed a further increase in liver enzymes, indicating a worsening of her shocked liver (Table 6). Her platelets decreased, the international normalized ratio increased, the fibrin degradation product increased, and fibrinogen was in a non-critical range (Table 7). A diagnosis of disseminated intravascular coagulation was made, and labs were trended. Significant purpura developed in the patient’s left leg in the following days.

**Table 6:** Liver enzymes from day two of hospital admission

Labs	Patient lab values	Normal reference lab values
Aspartate transferase	2,754 U/l	9-40 U/l
Alanine transaminase	3,589 U/l	7-60 U/l

**Table 7:** Relevant labs for clotting and bleeding risk on day two of hospital admission

Labs	Patient lab values	Normal reference lab values
Platelets	62 K/mcl	150-400 K/mcl
International normalized ratio	3.80	0.8-1.2
Fibrin degradation product	>20.00 mg/l	0.50 mg/l
Fibrinogen	437 mg/dl	200–400 mg/dl

The remainder of the patient’s hospital course was unremarkable. She remained in the hospital for 14 days to complete the course of ceftriaxone. After completing her course of antibiotics, she was discharged in stable condition.

## Discussion

Increased rates of infection due to immunosuppression is a well-established concept. Increased rates of *N. meningitidis* in immunocompromised patients is also a concept that has been introduced previously. In a study of patients who had received a solid organ transplant, it was shown that there was a seven-fold higher incidence of bacterial meningitis when compared to the general population [6]. A prospective cohort study on the effects of adjunctive dexamethasone and the prognosis of bacterial meningitis showed that 6% of the 1,447 cases of community-acquired bacterial meningitis occurred in patients receiving immunosuppressive medications [7]. Both of these studies also demonstrated that immunosuppressed patients were less likely to display typical symptoms of bacterial meningitis, such as headache and neck stiffness when compared to the general population [6,7].

Eculizumab and ravulizumab are two medications that are classically associated with an increased risk of *N. meningitidis*. Both are monoclonal antibodies that have been approved for the treatment of paroxysmal nocturnal hemoglobinuria and atypical hemolytic uremic syndrome. They work by inhibiting the cleavage of C5 into C5a and C5b [8,9]. This inhibits complement pore formation and hinders the body’s ability to defend against encapsulated bacteria. Due to this, eculizumab and ravulizumab both carry a black box warning for an increased risk of life-threatening and fatal meningococcal disease [8,9].

The novelty of this case is that this is a presentation of *N. meningitidis* in a patient immunosuppressed due to adalimumab. A single previous case report described a case of subacute meningococcaemia in a patient taking adalimumab [10]. However, this case shows a much more severe and life-threatening presentation. Adalimumab is an IgG monoclonal antibody that binds to TNF-alpha, neutralizes its bioactivity, and induces apoptosis of TNF-expressing mononuclear cells [11]. The TNF-alpha is an inflammatory cytokine that contributes to cell signaling leading to necrosis and apoptosis [12]. Inhibiting TNF-alpha decreased the patient's innate immune response and made her more susceptible to infection. Currently, adalimumab is indicated for rheumatoid arthritis, juvenile idiopathic arthritis, psoriatic arthritis, ankylosing spondylitis, Crohn’s disease, ulcerative colitis, plaque psoriasis, hidradenitis suppurativa, and uveitis [11,13]. The patient in this case was prescribed adalimumab to treat severe hidradenitis suppurativa. It was also the most likely cause of her lack of headache and lack of nuchal rigidity.

Medical staff should remain vigilant in patients who are taking immunosuppressive therapy. The lack of typical symptoms such as headache and nuchal rigidity does not rule out a serious and life-threatening diagnosis such as *N. meningitidis*. Prompt recognition of immunosuppressive medications in a patient’s chart should be encouraged by all medical staff. This could lead to a broader differential and more thorough workup, allowing the etiology of the patient’s symptoms not to be missed. The risk of infection should be discussed in length with patients before initiating immunosuppressive therapy. Preventative measures such as vaccination should also be routinely discussed with patients on immunosuppressive therapy.

## Conclusions

This case illustrates a presentation of N. meningitidis. The most likely cause of the patient’s infection and symptomatology was the use of adalimumab. This report highlights the importance of maintaining a broad differential, the need for a thorough diagnostic workup in a patient on immunosuppressive therapy, and vaccinating patients before initiating immunosuppressive therapy.
